# Associations between accessibility to health care service, social support, and Korean Americans’ mental health status amid the COVID-19 pandemic

**DOI:** 10.1186/s12889-021-11820-7

**Published:** 2021-10-27

**Authors:** Jihyun Jane Min, Shinwoo Choi, Hyejoon Park

**Affiliations:** 1 Krieger School of Arts and Sciences, Johns Hopkins University, MD 21218 Baltimore, USA; 2 School of Social Work, Texas State University, San Marcos, TX 78666 USA; 3 School of Social Work, Western Michigan University, Kalamazoo, MI 49007 USA

**Keywords:** COVID-19, Mental health, Korean Americans, Psychological distress, Accessibility to health care service, Language barrier, Family social support

## Abstract

**Background:**

While previous studies have examined the relationships between social support and health care accessibility among ethnic minority populations, studies on Korean Americans remain scarce. Therefore, this study aims to assess the relationship between Korean Americans’ mental health, accessibility to health care, and how they perceive the level of social support during the COVID-19 pandemic.

**Method/result:**

We distributed online surveys to Korean Americans from May 24, 2020, to June 14, 2020, generating 790 responses from participants residing in 42 states. Binary Logistic and Ordinary Least Square regression analyses revealed that poor mental health was associated with language barriers inhibiting Korean Americans’ access to COVID-19-related information. Their perceived social support from family members and close friends was positively associated with mental health.

**Conclusion:**

Our findings recommend that equipping community health care services with translators or interpreters is necessary. Additionally, health practitioners and staff should be trained to utilize telehealth tools to effectively treat individuals with mental health problems. American policymakers and health care professionals need to understand and address the unique hardships Korean Americans experience amid COVID-19.

## Introduction

Since the outbreak of Coronavirus Disease 19 (COVID-19) from Wuhan, the Republic of China, on December 31, 2019 [1], this virus has spread to more than 114 countries [2]. Responding to the rapid growth of COVID-19 cases, many nations, including the U.S. have attempted to prevent the pandemic by putting extensive protection measures in place for the safety of their citizens. These include the limitation of free individual travel, maintaining a six-foot social distance, regulating face-to-face educational and public activity, and limiting access to leisure, sporting activities, and religious ceremonies (e.g., worship, funerals) [2].

Most recent studies have already noticed that the COVID-19 pandemic not only negatively impacts individuals’ physical health but also mental health [3–5]. For instance, Holmes et al. (2020) [3] discovered that COVID-19 affected individuals’ quality of mental conditions, increasing levels of stress and harmful behaviors, such as suicide and self-harm, while coping with extraordinary circumstances. Li et al. (2020) [4] also determined that during the health emergency due to COVID-19, many Chinese residents living or traveling near epidemic areas identified increased internalized stigmatization or mental health problems, such as low levels of self-worth or depression. On top of that, many Asian Americans have already reported that wearing masks increased the perceived levels of racial discrimination they experienced, accelerating their levels of depressive symptoms [5].

Therefore, it is crucial to understand cumulated social risks and locate preventative factors to expand effective public health practices and reduce health disparities predominantly among racial/ethnic minority groups in the U. S [6]. For this purpose, it is worth discerning positive factors to assist Korean Americans—who make up one of the top five origin countries of Asian immigrants in the U.S. [7] but whose health has been understudied and treated as a subgroup over Asian Americans (particularly, Chinese)—in coping with significant social risk factors (e.g., pandemic) and reducing their mental health crisis in an urgent situation. This will support the U.S. public health system to effectively apply their practice to Korean Americans who are likely to experience high levels of mental health problems.

For this study—examining the connection between accessibility to community services, as well as social support and mental health conditions of Korean Americans amid COVID-19—firstly, we deliberated the historical background of Korean Americans, social risk factors as to racial discrimination against Asians and their mental health, the role of community health care service and family social support for racial/ethnic groups’ mental health, and mental health during COVID-19 in the literature review. Secondly, we explained our study’s method and results with the conduction of a primary study by collecting datasets from Korean Americans. Lastly, we discussed our study and suggested several solutions for how the public and community health practitioners could play their roles effectively to promote the psychological well-being of Korean American communities amid the COVID-19 pandemic.

## Literature review

### Past and present situations of Korean Americans

From 1903 to 1905, 7000 Koreans were moved to Hawaii to work as plantation laborers due to the labor demand on Hawaiian plantations after the Chinese labor immigration act [8]. Since the 1960s, the Korean American population has dramatically increased as a result of political, economic, and military relations between South Korea and the U.S. In 2017, about one million Korean immigrants resided in the United States [9].

As of 2010, the racial population percentage of the U.S. is 64.7% White, non-Hispanic, 16% Hispanic or Latino, 13.6% Black, 5.3% Asian, and 1.8% Multiracial groups. This percentage is predicted to change to 46.3, 30.2, 15.0, 9.2, and 3.9% respectively by 2050 [6]. Out of the Asian population, the Korean group is ranked as the fifth-largest after India, China, the Philippines, and Vietnam [7]. According to the percentage change, it is noticeable that racial/ethnic groups other than White, non-Hispanic will continue to grow, and they will take up more than 50% of the U.S. population.

Notably, Korean Americans are likely to have higher incomes and educational achievement and less likely to experience poverty or lack health insurance. In 2017, the median income of Korean Americans’ households was $65,000 compared to $57,000 for all immigrant households and $61,000 for U.S.-born families; around 34% of Korean Americans had a bachelor’s degree, compared to 18% of the foreign-born and 20% of the U.S.-born populations; and 20% of Korean Americans had graduate or professional degrees compared to other immigrants (13%) and native-born Americans (12%). However, Korean Americans have lower workforce participation rates and lower English proficiency than other racial/ethnic immigrant populations [9].

### Korean Americans’ experiences of mental health

The U.S. government has made an effort to reduce racial discrimination and promote economic and social justice in various sectors, such as profit- or non-profit, the government, and the educational sector. However, in spite of their efforts, fully exerting racial equality is difficult to do in reality. Even though the U.S. is considered a “majority-minority” nation, non-White racial/ethnic groups are disproportionally represented among those living in poverty and child welfare services and experience racial disparities in employment, health care access, and schools; furthermore, communities are visibly segregated [6].

Racial discrimination affects notably mental and physical health among minority populations [10–12]. However, so far, the study of racial discrimination among racial/ethnic groups has heavily focused on Black communities, and it is urgent to extend the scope of this subject to other minority groups, including Asians [13, 14].

A study by Noh et al. (2007) [15], one of few studies that examined the impact of racial discrimination on the mental health of Korean Americans, discovered that experiencing subtle bias or unfair treatment (e.g., perceived discrimination) caused Korean Americans to produce distress symptoms because of their unstable or ambiguous social identity.

### Mental health and accessibility to health care services in immigrant populations

Many immigrants experience more mental health problems than native-born individuals. However, unfortunately, they are less likely to use health care services for mental illness and experience barriers—in particular, language difficulties and lack of knowledge about the available resources—when trying to access care for mental health problems [16]. Such physical hardships could exacerbate immigrants’ mental health problems. On the other hand, the longer the migrants (called health immigrants) are in the U.S., the more likely they are to access health care services [17]. Their study implies that language barriers and lack of information about their communities could be significant factors that hamper immigrants’ access to health care services in communities.

It has been previously noted that English fluency affects immigrants’ utilization of health care services. Non-U.S.-born immigrants are prone to underuse health care services, heavily rely on ‘ad hoc interpreters’ (family members, friends, relatives) when seeing health professionals, have less understanding of the care they receive, have delayed diagnoses, be less satisfied with the care they receive, and be less likely to follow recommendations for treatment [16, 18–20]. To meet the needs of those immigrants, communities that have high immigrant populations are recommended to employ translators or interpreters whose assistance these immigrant patients can easily ask for whenever they visit community health clinics or services. In addition, training staff to work with non-English speaking immigrants and preparing enough resources written in different languages are essential to take care of these immigrants more effectively [21].

### Mental health and social support

It has been found that having greater social support—social resources that individuals perceive as available, either instrumental/tangible in a physical way or emotional connectedness, optimism, and adaptive coping responses from family members—assists in lowering an individual’s mental health problems, including chronic stress and depression, and optimizes psychological well-being [20, 22]. On the other hand, a study by Chou et al. (2011) also verified that negative social support, such as the absence of frequent contact with close friends, family members, and religious groups, was associated with mental health issues (e.g., depression, multiple moods anxiety, and substance use disorders) [20, 23]. More specifically, infrequent religious contact was significantly related to substance use disorders; infrequent meetings with close friends and family members were significantly associated with major depressive disorder [24].

Additionally, Smyth et al. (2014) pointed out that having a high level of emotional support was associated with a decreased common mental disorder, and having weekly family and friend contact was associated with a decrease of depressive episodes. Overall, emotional support, social support, and contact with family and friends were more crucial protective factors for a range of mental health outcomes [23].

### Mental health and social support in racial/ethnic populations

Social support and social networks were demonstrated to be effective in particular for the mental health of ethnic minority groups who value family, kin, and culture highly [23, 25, 26]. This situation is more evident in households whose primary language is different from English. Almedia et al. (2009) observed that the Mexican participants whose mother tongue is not English and are not U.S.-born had more frequent family connections and support than U.S.-born generations whose primary language is English. The reason why foreign-born Mexicans had close contact with family members and received family support was that they had strong traditional cultural values and acculturation, and such support could be a healthy advantage. At the same time, they could not rely on non-kin social ties due to migration [26].

However, even though numerous studies noticed the variation of social support across population subgroups [23, 25–27], few studies have aimed to explore the association between social and family support and Asian communities, including Korean families [26]. The most recent studies by Liu et al. (2020) [5], Li et al. (2020) [4], and Choi et al. (2020) [28]—all of which investigated East Asians’ mental health, social support, mental health amid COVID-19—discovered that, even though all participants in the studies reported high levels of psychological distress, Li et al. (2020) [4] did not find a significant association between perceived support and a decrease of mental health problems, while Choi et al. (2020) [28] unearthed that Koreans’ psychosocial distress due to racial discrimination during the COVID-19 pandemic was likely to be reduced because of overall social support. 

### Mental health, health care services, and COVID-19

The outbreak of COVID-19 has caused individuals not only to experience physical threats but also to increase psychological and mental problems [29, 30]. According to a recent survey in the U.S. Health Affairs, the elderly reported that stress from COVID-19 had been a challenge, while a third of Americans reported mental health problems [30]. Rapid contagious disease resulted in lockdowns in many households and communities, which in turn led them to experience drastic social distancing, self-quarantine, another disease, isolation, xenophobia, and mental health illness, such as depression and stress [29]. Most notably, quarantine and self-isolation prevalently cause individuals to have a severe mental illness. The separation from loved ones, loss of freedom, boredom, and uncertainty most likely affect individuals by worsening their mental status [29]. In addition, a few studies have just discovered that many Asian Americans (Chinese, Koreans) appealed that they also have high levels of psychosocial distress due to perceived racial discrimination amid the COVID-19 pandemic [5, 28].

To alleviate mental problems considering the pandemic situation, virtual communication and supportive therapy were very effective for patients. The counselor utilized video consultations, the assigning of daily chores schedules, cognitive behavior therapy, and the establishment of phone contact with the elders and their family relatives. For many of her patients, these types of resources have led to reports of less compulsive behavior, instillation of hope, and reduction of apprehension. This more non-traditional form of health care access has decreased the mental health struggles that elders faced during the midst of the pandemic [30].

Another form of health care access that has shown significant improvements in the mental health of individuals was practicing yoga in India—a therapeutic intervention that included non-rigorous exercise, medication, and breath control techniques [31]. Along with its physical benefits, yoga has been shown to lighten an individual’s mood and decrease anxiety and depression among the elderly. With more accessibility to a health service, including a yoga intervention, elders’ mental health has improved drastically [31].

## Materials and method

### Data procedure

Korean Americans above the age of 18 residing in the U.S. (including both U.S.-born and foreign-born) were recruited by purposive sampling from May 24, 2020, to June 14, 2020. The respondents were invited to participate in an online survey to study Korean Americans’ well-being during the COVID-19 pandemic. The invitations were distributed by emails and postings on Korean Americans’ online communities. There were two language versions, English and Korean, on the online survey. 90% of participants selected the Korean version. Although relying on a non-probability sampling strategy reduces the representativeness of the sample [32], it was the most feasible method for the current study due to timing and feasibility issues. It is mainly because a sampling frame does not exist for the specific target population: the entire Korean American population currently residing in the U.S. For instance, it was estimated that there were 1.8 million Korean Americans in 2015 [33]. If there is a sampling frame (i.e., a list of the entire 1.8 million Korean Americans in the U.S. and their contact information), applying one of the random sampling methods will yield a more representative sample.

Moreover, without the sampling frame and the time-sensitive nature of the research questions, the authors reached a decision that relies on a purposive sampling strategy that will be a good fit amid the COVID-19 pandemic. It is noted that a non-random sampling strategy can still provide meaningful information in a timely manner [32]. Relying on email distributions and postings on Korean American online communities has some limitations. However, it is still one of the widely used methods to recruit the target respondents [34].

### Measurements

A research team composed of five multicultural and bilingual social science scholars selected all the questions that were included in the survey. For the main variables, existing scales that are reliable and valid were chosen. The scales were first translated from English to Korean. Afterward, it was back-translated into English in order to increase the rigor of the test items. For some items that did not have any existing scales, the research team developed the items in both languages. In order to make sure that the items were both linguistically and culturally conveying the correct messages, reiterative processes assessed the quality of the items in the survey.

*Self-rated mental health* was measured by a single item that asked the respondents’ overall mental health condition over the last 30 days (1 = good, 0 = poor). Despite having only a single item, it is moderately correlated with other scales measuring psychological status and is widely used [35, 36].

*Psychological distress* was measured using a scale with high reliability of .93 [37]. The Kessler Psychological Distress Scale (K10) asks the participants to describe their psychological functioning over the last month. The ten items measure individuals’ specific distress levels, including their anxiety, depression, and psychological distress. Items such as “About how often did you feel nervous?” or “About how often did you feel so sad that nothing could cheer you up?” are in the scale. The ten items are supposed to measure the “frequency of non-specific psychological distress symptoms” during the previous month [38]. It is well correlated with and a sensitive screen for the Diagnostic and Statistical Manual of Mental Disorders’ criteria for mood disorders and anxiety [38]. Respondents indicated their answers on a 5-point Likert scale from 1 (none of the time) to 5 (all the time). A total score (range 10–50) was used as a continuous variable in the data analysis process.

*Accessibility of health care resources* was measured by five items related to individuals’ accessibility to receive health care during the COVID-19 pandemic. The items asked whether the individuals had health care coverage, if they had primary health providers, if they knew where to get tested for COVID-19, if they were experiencing language barriers when trying to obtain information on COVID-19, and if they were willing to receive mental health services if needed. The questions were asked in a dichotomous format (1 = yes, 0 = no).

*Social support* was measured by using the three items from the Social Interaction Scale [39].

The scale measures (1) how much the respondent can confide in close ones (e.g., family, relatives, or friends) to talk about worries, (2) how much the respondent can rely on close ones (e.g., family, relatives, or friends) to help with a serious problem, and (3) how much the respondent contacts family members and close friends. The three items from the scale were used to measure the respondents’ perceived level of social support that they will receive in a difficult situation. The responses can range from (1 = not at all) to (4 = a lot). A total score (range 3–12) of the three items was used as a continuous variable for data analysis purposes.

*Sociodemographic factors.* Gender, age, years of education, household income, and employment status were controlled for analysis in order to examine the effects of accessibility to community health care and social support on individuals’ mental health outcomes.

### Data analysis

SPSS 24.0 was used for data analysis. List-wise deletion was used for missing values if the remaining cases were large enough. After the data collection, there were 959 Korean Americans who responded to the survey. After employing list-wise deletion to take care of missing values, there were 790 people who remained in the final sample (82% of the initial sample). First, in order to find out the distribution of all the variables, descriptive statistics were employed. Afterward, a binary logistic regression was conducted in order to test the effect of accessibility to health care services and social support on the individual’s self-rated mental health status. Finally, Ordinary Least Squares (OLS) regression was conducted in order to examine the impact of accessibility to health care services and social support on Korean Americans’ level of psychological distress. Prior to conducting any regression analyses, multicollinearity and collinearity were ensured for the variables included in the models.

## Results

### Findings

Table 1 shows the sample characteristics of the final sample (*N* = 790). The mean age was 42.74 (range: 20–81). 60% were female, and 40% male. For educational background, 8% had at least a high school diploma, 21% had some level of college education, 34% had a bachelor’s degree, and the other 37% had a graduate degree. In terms of current employment status, 14% were employed part-time, 53% were employed full-time, and the other 32% were not in the labor force. 21% of the sample had below $34,999 as their annual household income, 13% earned $34,999- $49,999, 38% earned $50,000- $99,999 and the other 28% earned above $100,000 per year. In terms of self-rated mental health status, more than half of the sample (72.6%) reported that their mental health status was poor. The other (27.4%) had good mental health.

**Table 1:** Characteristics of the Study Sample

Mean or percentage			
Variables	All sample (***N*** = 790)	Variables	All sample (***N*** = 790)
**Dependent Variable**		**Control variables**	
***Self-rated mental health***		***Education***	
Poor	72.6%	High school diploma or less	7.9%
Good	27.4%	Some level of college education	20.9%
***Psychological distress***		Bachelor’s degree	34.4%
Min:10	*Mean: 18.95*	Graduate degree	36.8%
Max: 49	Std.Dev:7.05	***Employment status***	
**Independent Variables**		Employed full time	53.4%
**Accessibility to health care services**		Employed part-time	14.3%
***Health insurance***		Out of labor force	32.4%
Yes	86.8%	***Household income***	
No	13.2%	< $34,999	21.6%
***Primary health provider***		$35,000–$49,999	13.3%
Yes	62.4%	$50,000- $99,999	27.8%
No	37.6%	> = $100,000	27.8%
***COVID-19 test knowledge***		***Sex***	
Yes	62.4%	Male	40.3%
No	37.6%	Female	59.7%
***Mental health service willing***		***Age (years)***	
Yes	83.2%	Min: 20	Mean: 42.74
No	16.8%	Max: 80	Std.Dev: 10.96
***Language barrier COVID-19***			
Yes	27.3%		
No	72.7%		
**Social support**			
Min:3	Mean:12.04		
Max: 15	Std.Dev:2.44		

Table 2 presents a binary logistic regression model, which was estimated to address the first research question: “Are accessibility to health care services and social support related to Korean Americans’ mental health status amid COVID-19?” This logistic regression examined how the effect of five types of accessibility to health care and social support contributed to the probability of having good or poor self-rated mental health while controlling for respondents’ socioeconomic and demographic characteristics.

**Table 2:** Binary Logistic Regression Model on Self-Rated Overall Mental Health

Korean Americans amid COVID-19 (***N*** = 790)			95% CI
Variables	Coeff. (S.E)	Odds Ratio	
**Accessibility to health care services and social support**			
Health insurance	.293	−.028	[.548, 1.727]
Primary health provider	.211	−.167	[.559, 1.280]
COVID-19 test knowledge	.363	−.150	[.571, 2.361]
Mental health service willing	.242	−.021	[.610, 1.573]
Language barrier COVID-19	.242***	−.644	[.327, .844]
Social support	.043****	.171	[1.091, 1.291]
**Individual Characteristics**			
Age	.009**	.017	[1.000, 1.036]
Sex (Male)			
Female	.201	−.700	[.335, .737]
Years of Education (Graduate)			
High school diploma	.360	.409	[.744, 3.047]
Some college	.198	−.012	[.670, 1.456]
Household Income (< $100,000)			
< $34,999	.291	−.214	[.457, 1.427]
$35,000 - $49,999	.315	.022	[.551, 1.894]
$50,000 - $99,999	.227	−.039	[.616, 1.501]
Employment Status (Full-time)			
Part-time	.302	−.063	[.519, 1.698]
Out of labor force	.229	−.077	[.519, 1.698]
Constant	.838****	−3.246	

***Note.*** Categories in parentheses are reference groups

For the Model, **p ≤. 05. ***p ≤ .01 ****p ≤ .001

The association between Korean Americans’ experience of a language barrier while trying to receive up-to-date information about COVID-19 and their self-rated mental health status was negative and statistically significant after controlling the effects of demographic factors. Those who reported that they experience language barriers were less likely to report having a good self-rated mental health status (OR = −.644, *p*  .05; 95% CI [.327, .844]). Furthermore, Korean Americans with higher social support were more likely to have higher self-rated mental health (OR = .171, *p*  .001; 95% CI [1.091, 1.291]).

However, the other variables related to individuals’ accessibility to health care services (e.g., having health insurance, having a primary health provider, knowing where to get tested for COVID-19, and willingness to receive mental health services if needed) did not show significant associations. In terms of demographic variables, age (OR = .017, *p*  .05; 95% CI [1.000, 1.036]) showed a significant relationship with self-rated mental health.

Table 3 presents the OLS regression model, which was estimated to answer the second research question: “Are accessibility to health care services and social support related to Korean Americans’ level of psychological distress amid COVID-19?” This regression model examined how accessibility to community health care services as well as the level of social support affected their level of psychological distress. Consistent with the first binary logistic regression model on Korean Americans’ self-rated mental health, the second regression model found a similar pattern.

**Table 3:** OLS Regression Model on Psychological Distress

Korean Americans amid COVID-19 (*N* = 790)			95% CI
Variables	B	S.E(B)	
**Accessibility to health care services and social support**			
Health insurance	−.761	−.036	[−2.375, .852]
Primary health provider	.618	.042	[−.575, 1.810]
COVID-19 test knowledge	−1.156	−.046	[−2.994, .681]
Mental health service willing	.318	.017	[−1.034, 1.671]
Language barrier COVID-19	1.659***	.103	[.433, 2.885]
Social support	−.727****	−.246	[−.945, −.509]
**Individual Characteristics**			
Age	−.090	−.146	[−.140, −.040]
Sex (Male)			
Female	3.488	.240	[2.354, 4.621]
Years of Education (Graduate)			
High school diploma	−.665	−.025	[−2.666, 4.621]
Some college	−.641	−.043	[−1.734, .452]
Household Income (< $100,000)			
< $34,999	1.240	.072	[−.339, 2.819]
$35,000 - $49,999	−.157	−.007	[−1.926, 1.612]
$50,000 - $99,999	.710	.048	[−.589, 2.008]
Employment Status (Full-time)			
Part-time	−.079	−.004	[−1.706, 1.548]
Out of labor force	.614	−.004	[−.627, 1.855]
F (df)	8.673 (15)	.0000	

***Note***. Categories in parentheses are reference groups

For the Model, ***p ≤ .01 ****p ≤ .001

Considering the accessibility to health care service variables, respondents’ experience of language barriers while trying to gain information about COVID-19 was significantly associated with the level of psychological distress (B = 1.659, *p* .01; 95% CI [.433–2.885]). Likewise, the level of perceived social support was also significantly associated with the level of psychological distress (B = −.727, *p* .001; 95% CI [−.945, .509]).

## Discussion

The fact that a majority of Korean Americans (72.6%) in our sample reported poor mental health status amid COVID-19 is alarming. This finding is consistent with the previous study by Choi et al. (2020) [28], albeit quite surprising. Still, considering the relatively higher socioeconomic status (SES) in our sample, it is understandable that there was no correlation between having health insurance and poor mental health. As O’Connor and Batalova (2019) described in their report, many Korean Americans have the financial means to get private insurance [9]. The majority of our sample had health insurance coverage, primary health providers, and easy access to information on COVID-19. The fact that they are willing to receive mental health services if needed is also a good signal that this population is likely to access appropriate professional services.

However, the experience of a language barrier while trying to receive up-to-date information on COVID-19 was significantly associated with Korean Americans’ mental health. This finding is supported by numerous studies indicating that, due to the lack of English fluency, many non-U.S.-born Korean Americans have difficulties and barriers to obtaining health-related information or accessibility to utilize health care services in their communities. This experience would severely affect Korean Americans’ mental status [16–19].

With respect to social support in our study, whether Korean Americans were in close relationships and contact with their family and friends was significantly associated with their mental health status. This finding is also consistent with the previous studies, which unveiled the important role that social support plays in individuals’ mental health [20, 26, 28, 40]. This result is also understandable since many Korean Americans compose nuclear families in the U.S., and the study took place amid COVID-19 when having conversations with them by phone or email can be the best avenue for them to share their concerns, worries, and updates. In addition, valuing family highly is the accepted norm in Asian culture. Korean Americans’ frequent contact with their families and close friends helps them keep this cultural value, which in turn, could help them to cope with the stressful COVID-19 situation.

We used two different outcome measurements to assess Korean Americans’ mental health status amid the COVID-19 pandemic. Even though the scale system was different, our project emphasized rigorous outcomes indicating Korean Americans significantly and highly experienced negative mental health as well as its psychometric level of anxiety, depression, and psychological distress during COVID-19 based on the accessibility to health care services (e.g., language barriers) and social support (e.g., close relationships and contacts with family members and friends).

### Implications

Individuals’ mental health status is concerning since the pandemic continues [5, 28, 29, 41]. In order to protect individuals’ vulnerable health during this time, preventive mental health programs should be widely available for diverse populations [24, 26]. In particular, considering the pandemic, utilization of Telehealth, a remote service, should be more common [42]. This is helpful for individuals with health problems or elderly individuals who are in a self-quarantine situation and, therefore, barely access health care services from nearby community facilities. To facilitate the instrument, community hospitals, health clinics, government-funded health agencies, and private practitioners need to be informed how to use the internet or telephone to properly communicate and interact with patients, and they should be trained for Information Technology fluency. For instance, using Telehealth services and instructing therapeutic yoga would be ideal. This tool will be more effective in treating individuals with mental health problems and elderly individuals than face-to-face treatments amid COVID-19 [42].

Since Korean Americans reported that they experienced language barriers when trying to receive recent information on COVID-19, there should be a more active effort to provide important information to diverse populations. For instance, hiring Korean-English translators, bilingual practitioners, or contracting with a medical translation service in community clinics or the hospital is recommended. Furthermore, as Durbin et al. (2016) addressed already, preparing resources written in Korean and training staff and health practitioners on how to appropriately interact with Korean Americans would be beneficial for them to treat mental health problems [21]. Lastly, equipping mental health services in primary care centers (e.g., Primary Care-Mental Health Integration) would be an ideal suggestion to help them to save time and reduce difficulty in finding psychiatric doctors to treat patients’ mental health problems. Indeed, health care and community organizations need to learn Asian languages to assist Asian American subgroups [43].

Since social support from families and close friends significantly affects Korean Americans’ mental health status, they should be encouraged to keep close contact with their family members and friends virtually. Many individuals suffer from social isolation during the pandemic, and Korean Americans are often cut off from their traditional social support and ties from their home country. Therefore, they should be encouraged and supported to receive social support in virtual and audible (Korean translation tools) ways [28]. However, the advice mentioned above would not be appropriate to those families who may choose not to stay in close contact or relationships because their family relationships or friendships are not healthy or supportive. For these families, as aforementioned in the literature review part, virtual communication or video consultations with health counselors or practitioners as well as supportive therapy, in particular yoga, would be beneficial to be utilized primarily for elderly Korean Americans. In spite of the adversity caused by COVID-19, many community engagements have emerged for psychosocial support and sources of resilience [44]. For instance, for social support and mental health amid COVID-19, community staff reach out to the individual household to ask for basic needs and reduce their loneliness. In addition, many long-term care facilities provide personal consulting with family members to make sure that their patients are well connected with their family members and their professional care is being met well [44]. Applications for these instances can be of help for Korean Americans who have been isolated from other kinship families due to the pandemic.

### Limitations

Our study has some limitations which cause generalization issues because this study sample was not established randomly. Hence, it is hard to represent the entire Korean American population in the U.S. Furthermore, the majority of the participants who responded to our survey were first-generation, so it has a lack of second or third-generation Americans. Secondly, as found, more than half of the participants earned a higher annual income and attained higher education (bachelor’s degree or above). Hence, it is hard to capture the condition of more vulnerable populations, such as low-income people. Third, we did not collect surveys from Korean Americans younger than 18 years old; therefore, we do not know if young Korean Americans would have a similar experience as our samples. Additionally, the sample size of (*N*  = 790) has limited capability to present the overall experiences of Korean Americans amid the COVID-19 pandemic. In April 2021, another survey was conducted to estimate Korean Americans’ perceived racism 1 year after the beginning of the pandemic [45]. The sample size was *N*  = 1244, which also relied on a non-random sampling strategy via purposive online survey invitations. Both surveys and other studies that relied on non-random sampling strategies due to feasibility issues still provide meaningful and important information in a timely manner. Moreover, future studies may utilize a random sampling strategy with a bigger sample size in order to recruit a more representative sample of the Korean American population.

Fourth, we used cross-sectional data in which data collection took place one time during the pandemic in May 2020. We do not know the long-term effects of racial discrimination during the pandemic on the respondents’ mental health status. Fifth, the survey asked participants for their perceived mental status, not by professional diagnosis. Thus, it is difficult to assess medically how the COVID-19 pandemic objectively influences the condition of participants’ mental health. Finally, this study only focused on Korean Americans living in the U.S.; therefore, it would be difficult to replicate its outcomes in Korean Americans residing in other countries with different social and historical backgrounds.

## Conclusion

To our knowledge, this is one of few studies that has aimed to investigate the relationship between the mental health of Korean Americans and the role of health care service and social support during the pandemic in the United States. Even though we have several limitations, our study findings could have some insight into how community professionals need to effectively approach Korean Americans who experience mental health problems and to what extent American policymakers and health care professionals understand Korean Americans’ culture and the hardships caused by such a unique and unprecedented pandemic.

## Data Availability

The data generated and analyses during the current study are not publicly available as CONVERGE Working Group members of “Korean Families and COVID-19” have own agreement/contract to use the dataset for the purpose of publications before a new study of post-COVID-19 follows but are available from the corresponding and the second author on reasonable request.

## References

[1] Novel Coronavirus (2019-nCoV) Situation Report 1: World Health Organization; Available from: https://www.who.int/docs/default-source/coronaviruse/situation-reports/20200121-sitrep-1-2019-ncov.pdf?sfvrsn=20a99c10_4.

[2] Who Director-General’s Opening Remarks at the Media Briefing on COVID-19: World Health Organization. Available from: https://www.who.int/dg/speeches/detail/who-director-general-s-opening-remarks-at-the-media-briefing-on-covid-19%2D%2D-11-march-2020.

[3] Holmes EA, O'Connor RC, Perry VH, Tracey I, Wessely S, Arseneault L, et al. Multidisciplinary research priorities for the COVID-19 pandemic: a call for action for mental health science. Lancet Psychiatry. 2020;7(6):547–60. 10.1016/S2215-0366(20)30168-1. 10.1016/S2215-0366(20)30168-1 PMC7159850 32304649

[4] Li J, Liang W, Yuan B, Zeng G (2020). Internalized stigmatization, social support, and individual mental health problems in the public health crisis. Int J Environ Res Public Health.

[5] Liu Y, Finch BK, Brenneke SG, Thomas K, Thao P. Perceived discrimination and mental distress amid the COVID-19 pandemic: evidence from the understanding America study. Am J Prev Med. 2020;59(4):481–92. 10.1016/j.amepre.2020.06.007 PMC7336127 32829968

[6] Barusch A (2015). Foundations of social policy: social justice in human perspective.

[7] Zong JBJ (2016). Asian immigrants in the United States in 2014. Migration policy institute.

[8] Chang E. Korean Americans Asian-nation: the landscape of Asian America; 2003. http://www.asian-nation.org/korean.shtml.

[9] O’Connor A, Batalova J. Korean immigrants in the United States; 2019. https://www.migrationpolicy.org/article/korean-immigrants-united-states-2017

[10] Krieger N (2014). Discrimination and health inequities. Int J Health Serv.

[11] Krieger N, Smith K, Naishadham D, Hartman C, Barbeau EM (2005). Experiences of discrimination: validity and reliability of a self-report measure for population health research on racism and health. Soc Sci Med (1982).

[12] Taylor J, Turner RJ (2002). Perceived discrimination, social stress, and depression in the transition to adulthood: racial contrasts. Soc Psychol Q.

[13] Bulatao RA, Anderson NB. Understanding racial and ethnic differences in health in late life: A research agenda; National Academics press; 2004 20669466

[14] Noh S, Beiser M, Kaspar V, Hou F, Rummens J (1999). Perceived racial discrimination, depression, and coping: a study of southeast Asian refugees in Canada. J Health Soc Behav.

[15] Noh S, Kaspar V, Wickrama KAS (2007). Overt and subtle racial discrimination and mental health: preliminary findings for Korean immigrants. Am J Public Health (1971).

[16] Regan FR (2015). Primary care for limited English-speaking patients and parents. J Am Assoc Nurse Pract.

[17] Straiton M, Reneflot A, Diaz E (2014). Immigrants’ use of primary health care services for mental health problems. BMC Health Serv Res.

[18] Bowen S (2001). Language barriers in access to health care.

[19] Yeo S (2004). Language barriers and access to care. Annu Rev Nurs Res.

[20] Steptoe A, O'Donnell K, Marmot M, Wardle J (2008). Positive affect and psychosocial processes related to health. Br J Psychol.

[21] Durbin A, Sirotich F, Durbin J (2016). English language abilities and unmet needs in community mental health services: a cross-sectional study. J Behav Health Serv Res.

[22] Seeman TE (2016). Health promoting effects of friends and family on health outcomes in older adults. Am J Health Promot.

[23] Smyth N, Smyth N, Siriwardhana C, Siriwardhana C, Hotopf M, Hotopf M (2015). Social networks, social support and psychiatric symptoms: social determinants and associations within a multicultural community population. Soc Psychiatry Psychiatr Epidemiol.

[24] Chou K-L, Liang K, Sareen J (2011). The association between social isolation and DSM-IV mood, anxiety, and substance use disorders: wave 2 of the National Epidemiologic Survey on alcohol and related conditions. J Clin Psychiatry.

[25] RMJP (1993). In the barrios: Latinos and the underclass debate.

[26] Almeida J, Molnar BE, Kawachi I, Subramanian SV (2009). Ethnicity and nativity status as determinants of perceived social support: testing the concept of familism. Soc Sci Med (1982).

[27] Turner RJ, Marino F (1994). Social support and social structure: a descriptive epidemiology. J Health Soc Behav.

[28] Choi S, Hong JY, Kim YJ, Park H (2020). Predicting Psychological Distress Amid the COVID-19 Pandemic by Machine Learning: Discrimination and Coping Mechanisms of Korean Immigrants in the U.S. Int J Environ Res Public Health.

[29] Javed B, Sarwer A, Soto EB, Z-u-R M (2020). Impact of SARS-CoV-2 (Coronavirus) pandemic on public mental health. Front Public Health.

[30] Prina L (2020). Funders support mental health care: COVID-19 and before. Health Aff.

[31] Mohanty S, Sharma P. Yoga for infirmity in geriatric population amidst COVID-19 pandemic. Asian J Psychiatr. 2020;53:1–2. 10.1016/j.ajp.2020.102199 PMC7293528 32590140

[32] RAB E (2016). Essential research methods for social work.

[33] Asian alone or in any combination by selected groups 2015. Available from: https://archive.ph/20200214011110/https://factfinder.census.gov/faces/tableservices/jsf/pages/productview.xhtml#.

[34] Saleh A, Bista K (2017). Examining factors impacting online survey response rates in educational research: perceptions of graduate students. J MultiDiscip Eval.

[35] Kessler RC, Andrews G, Colpe LJ, Hiripi E, Mroczek DK, Normand SLT (2002). Short screening scales to monitor population prevalences and trends in non-specific psychological distress. Psychol Med.

[36] Kroenke K, Spitzer RL, Williams JBW (2001). The PHQ-9: validity of a brief depression severity measure. J Gen Intern Med.

[37] K10 and K6 Scales: National Comorbiity Survey. Available from: https://www.hcp.med.harvard.edu/ncs/k6_scales.php.

[38] Bougie E, Arim RG, Kohen DE, Findlay LC (2016). Validation of the 10-item Kessler psychological distress scale (K10) in the 2012 Aboriginal peoples survey. Health Rep.

[39] Schuster TL, Kessler RC, Aseltine JRH (1990). Supportive interactions, negative interactions, and depressed mood. Am J Community Psychol.

[40] Suls J, Bunde J (2005). Anger, anxiety, and depression as risk factors for cardiovascular disease: the problems and implications of overlapping affective dispositions. Psychol Bull.

[41] Gómez-Salgado J, Andrés-Villas M, Domínguez-Salas S, Díaz-Milanés D, Ruiz-Frutos C (2020). Related health factors of psychological distress during the COVID-19 pandemic in Spain. Int J Environ Res Public Health.

[42] Lepkowsky CM (2020). Telehealth reimbursement allows access to mental health care during COVID-19. Am J Geriatr Psychiatry.

[43] Zhang M, Gurung A, Anglewicz P, Baniya K, Yun K. Discrimination and stress among Asian refugee populations during the COVID-19 pandemic: evidence from Bhutanese and Burmese refugees in the USA. J Racial Ethn Health Disparities. 2021. 10.1007/s40615-021-00992-y. 10.1007/s40615-021-00992-y PMC7924016 33651371

[44] Brief P (2020). COVID-19 and the need for action on mental health: United Nations.

[45] Jang S (2021). More than half of the Korean American reported being racially discriminated.

